# Critical role of tristetraprolin and AU‐rich element RNA‐binding protein 1 in the suppression of cancer cell growth by globular adiponectin

**DOI:** 10.1002/2211-5463.12541

**Published:** 2018-11-12

**Authors:** Nirmala Tilija Pun, Amrita Khakurel, Aastha Shrestha, Sang‐Hyun Kim, Pil‐Hoon Park

**Affiliations:** ^1^ College of Pharmacy Yeungnam University Gyeongsan Korea; ^2^ Department of Pharmacology School of Medicine Kyungpook National University Daegu Korea

**Keywords:** adiponectin, AUF1, Bcl‐2, hepatic cancer, TTP

## Abstract

Adiponectin exhibits potent antitumor activities. Herein, we examined the molecular mechanisms underlying suppression of tumor growth by globular adiponectin (gAcrp). We demonstrated that gAcrp suppressed B‐cell lymphoma 2 (Bcl‐2) expression, an anti‐apoptotic gene, by inducing its mRNA destabilization, which was accompanied with a decrease in cell viability and increased caspase‐3 activity in hepatic cancer cells. In addition, gAcrp increased expression of tristetraprolin (TTP) and AU‐rich element RNA‐binding protein 1 (AUF1), which are mRNA stability regulatory proteins. Moreover, gAcrp‐induced suppression of Bcl‐2 expression was abrogated by knockdown of TTP or AUF1. These data indicate that gAcrp induces apoptosis of hepatic cancer cells by TTP‐ and AUF1‐mediated Bcl‐2 mRNA destabilization, and further suggest that TTP and AUF1 are novel targets mediating the antitumor activity of adiponectin.

**Abbreviations**
3′‐UTR: 3′‐untranslated region
adipoR1: adiponectin receptor type 1
adipoR2: adiponectin receptor type 2
AMPK: adenosine monophosphate‐activated protein kinase
ARE: A (adenylate) +U (uridylate)‐rich elements
AUF1: AU‐rich element RNA‐binding protein 1
Bcl‐2: B‐cell lymphoma 2
DMEM: Dulbecco’s modi?ed eagle medium
FBS: fetal bovine serum
gAcrp: globular adiponectin
GAPDH: glyceraldehyde‐3‐phosphate dehydrogenase
HepG2: human hepatoma cancer cells
HRP: horseradish peroxidase
IL‐3: interleukin‐3
MCF‐7: human breast cancer cells
MTS: 3‐(4, 5‐dimethylthiazol‐2‐yl)‐5‐(3‐carboxymethoxyphenyl)‐2‐(4‐sulfophenyl)‐2H‐tetrazolium
PBS: phosphate buffer saline
PPAR‐α: peroxisome proliferator‐activated receptor alpha
PVDF: polyvinylidene difluoride
qRT‐PCR: quantitative real‐time polymerase chain reaction
TTP: tristetraprolin

Adiponectin, the most abundant adipokine, modulates various biological functions. In addition to its critical roles in insulin sensitization and lipid metabolism 1, 2, accumulating evidence also suggests that adiponectin possesses potent antitumor properties that are implemented through various mechanisms, including cell cycle arrest and apoptosis in cancer cells 3, 4. However, underlying molecular mechanisms are not clearly understood.

B‐cell lymphoma 2 (Bcl‐2) family proteins, acting as master regulators of apoptosis, consist of a number of anti‐apoptotic and pro‐apoptotic genes, both of which share Bcl‐2 homology domains 5. Among the various Bcl‐2 family proteins, Bcl‐2 modulates mitochondrial membrane permeability by binding to pro‐apoptotic proteins, including Bax and Bak, which further prevents cytochrome c release and finally inhibits apoptosis 6. Previous studies have demonstrated that enhanced Bcl‐2 expression is required for the survival of renal epithelial stem cells during embryonic development 7. In addition, high expression levels of Bcl‐2 are observed in B‐cell chronic lymphocyte leukemia and in neoplastic cells showing defective programmed cell death 8, 9, collectively indicating that Bcl‐2 expression is crucial for development and growth of various types of tumors. It has been well documented that adiponectin inhibits growth of cancer cells through various mechanisms 3, 10. Recent studies have revealed that adiponectin decreases Bcl‐2 expression 4; however, the molecular mechanism by which adiponectin suppresses Bcl‐2 expression in cancer cells is not well defined.

B‐cell lymphoma 2 expression can be determined at both transcriptional and post‐transcriptional levels. It has been shown that Bcl‐2 mRNA is enriched in AU‐rich elements (AREs) and Bcl‐2 expression can be determined by its mRNA stability, which is regulated by mRNA‐binding proteins. Of the various mRNA‐binding proteins, Tristetraprolin (TTP) and AU‐rich element RNA‐binding protein 1 (AUF1) bind to AREs at 3′‐UTR of the target mRNA and cause destabilization by enhancing its susceptibility to the RNA degradation machinery, thus promoting degradation of the target mRNA 11, 12. It has been reported that induction of TTP and AUF1 results in Bcl‐2 mRNA destabilization and induces apoptosis in cancer cells 13, 14. Furthermore, globular adiponectin (gAcrp) decreased Bcl‐2 expression in macrophages, which contributes to the suppression of inflammatory mediators’ expression 15. Based on previous reports, it is well established that Bcl‐2 plays an important role in determination of apoptosis in cancer cells and its expression can be modulated by mRNA‐destabilizing proteins. However, involvement of the mRNA‐binding proteins in the modulation of Bcl‐2 expression and tumor growth by adiponectin has not been explored.

Thus, to better understand the molecular mechanisms underlying antitumor activity of adiponectin, we examined whether gAcrp modulates stability of Bcl‐2 mRNA in hepatic cancer cells and further investigated potential underlying mechanisms. In the present study, we have demonstrated for the first time that gAcrp causes destabilization of Bcl‐2 mRNA. Moreover, gAcrp‐induced Bcl‐2 mRNA destabilization is mediated, at least in part, via induction of TTP and AUF1.

## Materials and methods

### Materials

All the cell culture reagents, unless mentioned elsewhere, were obtained from HyClone Laboratories (South Logan, UT, USA). Recombinant human gAcrp was brought from PeproTech, Inc. (Rocky Hill, NJ, USA). The primary antibody against TTP was procured from Abcam Biotechnology (Cambridge, UK); Bcl‐2 and AUF1 were from Cell Signaling Technology, Inc. (Beverly, MA, USA); β‐actin was procured from Thermo Scientific, Inc. (Rockford, IL, USA). The secondary antibody conjugated with horseradish peroxidase (HRP; goat anti‐rabbit IgG) was obtained from Pierce Biotechnology (Rockford, IL, USA).

### Cell culture

Human hepatoma cancer cells (HepG2) and human breast cancer cells (MCF‐7) were purchased from American Type Culture Collection (ATCC, Rockville, MD, USA). HepG2 and MCF‐7 cells were routinely cultured in Dulbecco's modified Eagle's medium supplemented with 10% FBS, 1% penicillin–streptomycin.

### Cell viability assay (MTS assay)

Cell viability was determined essentially as described previously 3. Briefly, after treatment with gAcrp, cells were incubated with MTS (3‐(4, 5‐dimethylthiazol‐2‐yl)‐5‐(3‐carboxymethoxyphenyl)‐2‐(4‐sulfophenyl)‐2H‐tetrazolium) solution for 2 h at 37 °C. The cell viability was determined based on the conversion of MTS tetrazolium to formazan, which is generated by metabolically active cells. The resultant cell viability was assessed by measuring absorbance at 490 nm using Versamax microplate reader (Sunnyvale, CA, USA).

### Caspase‐3/7 activity assay

Caspase‐3/7 activity was measured using Caspase‐Glo 3/7 assay kit (Promega, Madison, WI, USA) according to the manufacturer's instructions. Briefly, after treatment with gAcrp, cells were incubated with a luminogenic substrate and caspase 3/7 activity was determined by measuring luminescence generated from the cleavage of luminogenic substrate Ac‐DEVD‐pNA, using a microplate reader (Fluostar Optima, BMG Labtech, Ortenberg, Germany).

### Quantitative PCR

Bcl‐2 mRNA levels were measured as described previously 15. Briefly, after treatments as indicated, cells were lysed using Qiagen lysis buffer and total RNA was reverse‐transcribed. cDNA amplification was then performed by LightCycler system (Roche Diagnostics, Rotkreuz, Switzerland), using absolute quantitative PCR SYBR green capillary mix (Thermo Scientific) at 95 °C for 15 min, 40 cycles of 95 °C for 15 s, 60 °C for 30 s, and 72 °C for 30 s. The relative mRNA expression levels of target genes were determined after normalization with values of glyceraldehyde‐3‐phosphate dehydrogenase (GAPDH). Sequences of the primers used for PCR amplification are shown in Table 1.

**Table 1:** Sequences of the primers used for quantitative RT‐PCR

Target gene	Primer	Nucleotide sequences
Bcl‐2	F	5′‐ATGTGAGTGGAGAGCGTCAA‐3′
	R	5′‐ACAGTTCCACAAAGGCATCC‐3′
AdipoR1	F	5′‐ACGTTGGAGAGTCATCCCGTAT‐3′
	R	5′‐TCTTGAAGCAAGCCCGAAAG‐3′
AdipoR2	F	5′‐AGCCTCTATATCACCGGAGCTG‐3′
	R	5′‐GCTGATGAGAGTGAAACCAGATGT‐3′
GAPDH	F	5′‐ACCACAGTCCATGCCATCAC‐3′
	R	5′‐TCCACCACCCTGTTGCTGTA‐3′

### Transfection with small interfering RNAs

Human hepatoma cancer cells were seeded at a density of 3 × 10^5 ^cells/35‐mm dish. After overnight incubation, cells were transfected with small interfering RNA (siRNA) of target gene or with scrambled control siRNA using HiPerFect Transfection Reagent (Qiagen, Hilden, Germany) according to the manufacturer's instruction. Gene‐silencing efficiency was monitored by western blot analysis or quantitative real‐time polymerase chain reaction (qRT‐PCR). siRNA duplexes used in this study were chemically synthesized by Bioneer (Daejeon, South Korea) and are listed in Table 2.

**Table 2:** Nucleotide sequences of small interference RNA used for transient transfection

Target gene	Primer	Nucleotide sequences
TTP	F	5′‐GAGCUAUGUCGGACCUUCU‐3′
	R	5′‐AGAAGGUCCGACAUAGCUC‐3′
AUF1	F	5′‐GAUUACUUUGCUGCUAGUU‐3′
	R	5′‐AACUAGCAGCAAAGUAAUC‐3′
AdipoR1	F	5′‐CUCAUCAGAUUUUCCAUGU‐3′
	R	5′‐ACAUGGAAAAUCUGAUGAG‐3′
AdipoR2	F	5′‐CAGCAAAAGGUGGGAUCUA‐3′
	R	5′‐UAGAUCCCACCUUUUGCUG‐3′

### Western blot analysis

Protein expression levels were measured as described previously 16. Briefly, after treatments as indicated, total protein was extracted using radio‐immunoprecipitation assay (RIPA) lysis buffer supplemented with Halt Protease Inhibitor Cocktail (Thermo Scientific). Equal amounts of protein were loaded, separated by SDS/PAGE, and then transferred to polyvinylidene difluoride membranes. The membranes were then incubated with 5% skimmed milk in PBS/Tween‐20 (PBST) for 1 h and incubated overnight with specific primary antibodies at 4 °C. Membranes were further incubated with the secondary antibody conjugated with HRP for 1 h and treated with chemiluminescent substrates (Thermo Fisher Scientific). The images of the blot were finally captured using Fujifilm LAS‐4000 mini image analyzer (Fujifilm, Tokyo, Japan).

### Measurement of Bcl‐2 promoter activity

Promoter activity of Bcl‐2 was determined by Dual Luciferase Reporter Assay Kit (Promega) according to the manufacturer's instructions. Briefly, cells were initially seeded in 24‐well plate at a density of 8 × 10^4^ cells/well and were cotransfected with control (pRL‐TK) and expression vector (pGL2/Bcl‐2) using Fugene HD Transfection Reagent (Promega). Cells were then treated with adiponectin for indicated time periods. Firefly (Bcl‐2 promoter) and Renilla (control) luciferase activities were measured by the Dual‐Luciferase Reporter Assay System (Promega) according to the manufacturer's instructions. Bcl‐2 promoter activity was calculated by the ratio of firefly luciferase to Renilla luciferase.

### Statistical analysis

Data were analyzed by one‐way analysis of variance (ANOVA) combined with Tukey's *post hoc* multiple comparison test in graphpad prism software version 5.01 (La Jolla, CA, USA). Values are expressed as mean ± SEM from at least three independent experiments. Differences between groups were considered significant at *P* value < 0.05 (*P < 0.05). Half‐life (*t*_1/2_) of Bcl‐2 mRNA was calculated by sigma plot software version 12.6 (Systat Software, Inc., San Jose, CA, USA) using the equation First Parameter Logistic obtained by linear regression of plot of percentage of Bcl‐2 remaining versus time. Values are expressed as mean ± SEM from three independent experiments.

## Results

### Globular adiponectin induces apoptosis and decreases Bcl‐2 expression in HepG2 cells

**Figure 1:**
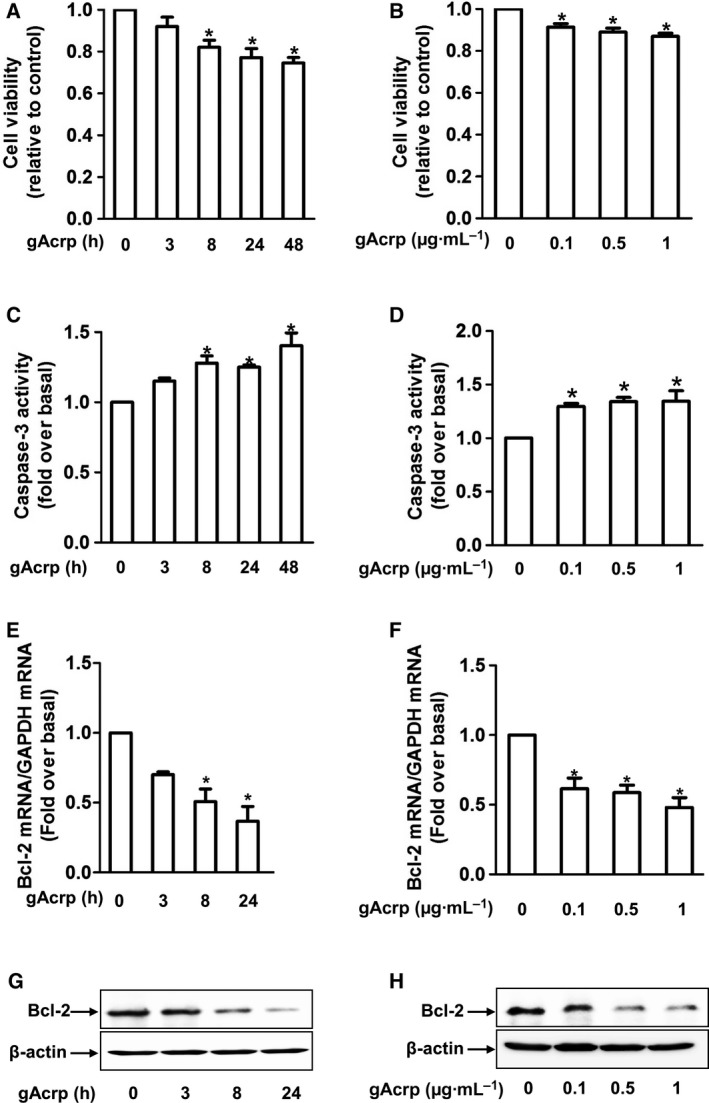
Effects of gAcrp on cell viability, caspase‐3 activity, and Bcl‐2 expression in HepG2 cells. HepG2 cells were treated with gAcrp (0.5 μg·mL^−1^) for the indicated time durations or indicated concentrations of gAcrp for 24 h. (A, B) Cell viability was determined by MTS assay. (C, D) Caspase‐3 activity was measured as described in methods. (E, F) Bcl‐2 mRNA expression level was assessed by qRT‐PCR. (G, H) Bcl‐2 protein expression was measured by western blot analysis. β‐actin was used as an internal loading control. Representative images from at least three sets of independent experiments are shown. Values represent fold increase in comparison with the control cells and expressed as mean ± SEM (*n* = 3–7). *P < 0.05 as compared to the control cells. Data in all figures (A–F) were analyzed by one‐way ANOVA, followed by Tukey's *post hoc* multiple comparison test.

To elucidate the mechanisms underlying suppression of cancer cell growth by adiponectin, we first confirmed the effect of gAcrp on cell viability in human hepatic cancer cells (HepG2). As shown in Fig. 1, gAcrp treatment significantly decreased cell viability in a time‐ (Fig. 1A) and dose‐dependent (Fig. 1B) manner consistent with previous reports (IC50 value is 4.88 ± 0.67 μg·mL^−1^). Decrease in cell viability by gAcrp was observed up to 72 h (Fig. S3), suggesting that suppressive effect of adiponectin on the growth of hepatic cancer cells would not be transient, but sustained long time duration. In addition, caspase‐3 enzyme activity was markedly increased in a time‐ (Fig. 1C) and dose‐dependent (Fig. 1D) manner. We also observed that gAcrp treatment had similar effects on cell viability and caspase‐7 enzyme activity in MCF‐7 breast cancer cells (Fig. S1A,B), collectively indicating that gAcrp suppresses growth of cancer cells via induction of apoptosis in our experimental conditions. In following experiments, we found that gAcrp prominently decreased Bcl‐2 mRNA expression in a time‐ and dose‐dependent manner (Fig. 1E,F). Essentially, similar effects on protein expression were also observed (Fig. 1G,H), implying that gAcrp causes apoptotic cell death in hepatic cancer cells by inhibiting expression of Bcl‐2, an anti‐apoptotic gene.

### Globular adiponectin induces Bcl‐2 mRNA destabilization in HepG2 cells

**Figure 2:**
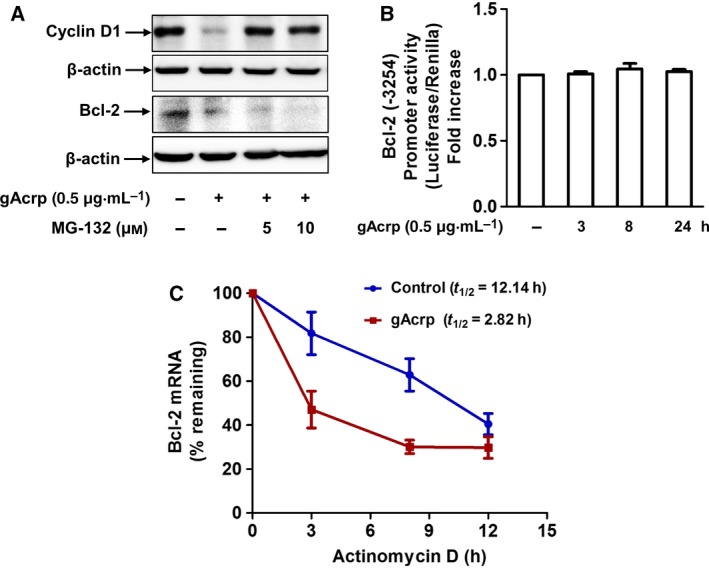
Modulation of Bcl‐2 mRNA stability by gAcrp in HepG2 cells. (A) HepG2 cells were pretreated with MG‐132, a pharmacological inhibitor of proteasome, for 2 h, followed by treatment with gAcrp (0.5 μg·mL^−1^) for additional 24 h. Bcl‐2 and cyclin D1 protein expression levels were determined by western blot analysis. Representative images from two sets of experiments are shown along with β‐actin as an internal loading control. (B) HepG2 cells were transiently cotransfected with the plasmid expressing pGL2/Bcl‐2 promoter and pTK‐RL (Promega), an expression vector for Renilla luciferase under the control of the thymidine kinase promoter, as an internal control reporter gene using Fugene HD transfection reagent (Promega) according to the manufacturer's instruction. After 24 h, cells were then treated with gAcrp (0.5 μg·mL^−1^) for the indicated time period. Firefly (promoter) and Renilla (control) luciferase activities were measured by the Dual Luciferase Reporter Assay System (Promega) according to the manufacturer's instructions. Bcl‐2 promoter activity was normalized to the relative activity of Renilla luciferase. Data were analyzed by one‐way ANOVA combined with Tukey's *post hoc* test, and values represent fold increase compared with control cells and are expressed as mean ± SEM (*n* = 3). (C) HepG2 cells were incubated with gAcrp (0.5 μg·mL^−1^) for 24 h. Cells were then treated with actinomycin D (2 μg·mL^−1^) up to 12 h. Messenger RNA levels of Bcl‐2 were measured by qRT‐PCR and normalized to GAPDH mRNA. Percentage remaining of Bcl‐2 mRNA was calculated as % of control (mean ± SEM,* n* = 3). Half‐life calculation and statistical significance were determined by sigma plot software version using equation First Parameter Logistic obtained by linear regression of plot from average mRNA % remaining and standard deviation.

We next investigated the mechanisms by which gAcrp suppresses Bcl‐2 expression. As Bcl‐2 expression levels can be determined at multiple stages, such as transcriptional, post‐transcriptional, and post‐translational levels, we first examined whether Bcl‐2 expression is regulated by proteasomal degradation. As shown in Fig. 2A, suppression of Bcl‐2 expression by gAcrp was not restored by pretreatment with MG‐132, a proteasome inhibitor, while MG‐132 treatment resulted in restoration of cyclin D1 expression, which was used as a positive control, indicating that proteasomal degradation might not be involved in the suppression of Bcl‐2 expression. To investigate whether gAcrp affects Bcl‐2 expression at transcriptional level, we analyzed the effect of gAcrp on Bcl‐2 promoter activity and observed that Bcl‐2 promoter activity, determined by luciferase reporter assay, was not significantly affected by gAcrp treatment (Fig. 2B). We finally tested whether gAcrp affects Bcl‐2 mRNA stability. For this, we examined the effect of gAcrp on half‐life of Bcl‐2 mRNA in the presence of actinomycin D, an inhibitor of *de novo* mRNA synthesis. As shown in Fig. 2C, gAcrp substantially decreased Bcl‐2 mRNA half‐life (12.14 h in the absence of gAcrp vs 2.82 h in the presence of gAcrp), clearly indicating that gAcrp causes destabilization of Bcl‐2 mRNA.

### Induction of TTP and AUF1 is involved in the suppression of Bcl‐2 expression by globular adiponectin in HepG2 cells

**Figure 3:**
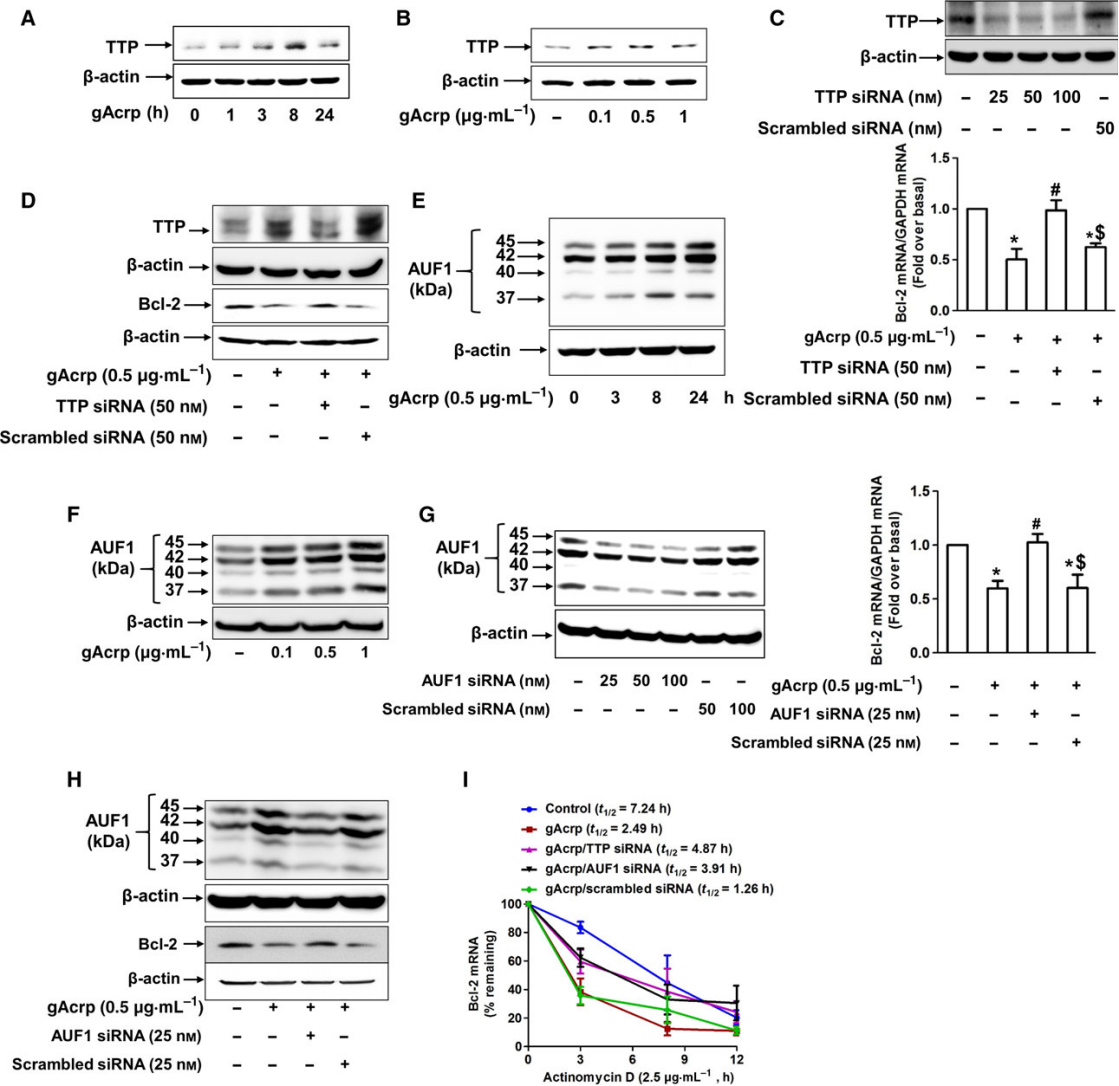
Role of TTP and AUF1 induction in destabilization of Bcl‐2 mRNA by gAcrp in HepG2 cells. (A, B) HepG2 cells were treated with gAcrp (0.5 μg·mL^−1^) for indicated time durations (A) or with indicated concentrations of gAcrp for 24 h (B). Protein expression of TTP was measured by western blot analysis. Representative images from three sets of independent experiments are shown along with β‐actin as an internal loading control. (C, D) HepG2 cells were transfected with siRNA targeting TTP or scrambled control siRNA followed by further incubation with gAcrp (0.5 μg·mL^−1^) for 36 h. TTP gene silencing was monitored by western blot analysis (upper panels in C). (C) Bcl‐2 mRNA level was measured by qRT‐PCR. Data were analyzed by one‐way ANOVA, followed by Tukey's *post hoc* test for multiple comparison, and values are shown as the fold changes relative to the control (fold over basal) and are presented as mean ± SEM (*n* = 5). *P < 0.05 compared to control cells; ^**#**^ *P* < 0.05 compared to the cells treated with gAcrp, but not transfected; ^$^ *P* < 0.05 compared to the cells treated with gAcrp and transfected with TTP siRNA. (D) Bcl‐2 and TTP protein expression was determined by western blot analysis. Representative images from three separate experiments are shown. β‐actin was used as an internal loading control. (E, F) HepG2 cells were treated with gAcrp (0.5 μg·mL^−1^) for indicated time durations (E) or with indicated concentrations of gAcrp for 24 h (F). AUF1 protein expression level was measured by western blot analyses. Representative images from three separate experiments are shown along with β‐actin as internal loading control. (G, H) Cells were transfected with AUF1 siRNA or scrambled control siRNA. After 36 h, cells were further stimulated with gAcrp (0.5 μg·mL^−1^) treatment for 24 h. Gene‐silencing efficiency was monitored by western blot analyses (upper panel in G). (G) Bcl‐2 mRNA level was measured by qRT‐PCR. One‐way ANOVA was used, followed by Tukey's *post hoc* multiple comparison test to analyze data, and values are shown as fold increases relative to the control and are indicated as mean ± SEM (*n* = 5). *P < 0.05 as compared to control; ^#^ *P* < 0.05 compared to the cells treated with gAcrp, but not transfected; ^$^ *P* < 0.05 compared to cells treated with gAcrp and transfected with AUF1 siRNA. (H) Bcl‐2 and AUF1 protein expression levels were determined by western blot analysis. β‐actin was used as internal loading control. (I) Cells were transfected with siRNA targeting TTP or AUF1 or scrambled control siRNA. After 36 h, cells were treated with gAcrp for 24 h, followed by treatment with actinomycin D (2 μg·mL^−1^) up to 12 h. Accumulating Bcl‐2 mRNA level was measured by qRT‐PCR and used for calculation of half‐life. Percentage remaining of Bcl‐2 mRNA was calculated as % of control (mean ± SEM,* n* = 3). Half‐life calculation and statistical significance were determined by sigma plot software version using equation First Parameter Logistic obtained by linear regression of plot from average mRNA % remaining and standard deviation.

In order to elucidate the mechanism underlying gAcrp‐induced destabilization of Bcl‐2 mRNA, we explored the role of TTP and AUF1, which act as mRNA‐destabilizing proteins. We first assessed the effects of gAcrp on the expression of TTP and AUF1. As shown in Fig. 3, gAcrp treatment increased TTP protein expression in a time‐ and dose‐dependent manner (Fig. 3A,B). In the following series of experiments to verify the functional role of TTP induction in the regulation of Bcl‐2 expression, we found that gene silencing of TTP restored the suppression of Bcl‐2 expression by gAcrp, at both mRNA (Fig. 3C) and protein (Fig. 3D) levels. Adiponectin also markedly increased AUF1 protein expression in a time‐ and dose‐dependent manner (Fig. 3E,F). In addition, gene silencing of AUF1 caused restoration of suppression of Bcl‐2 mRNA (Fig. 3G) and protein (Fig. 3H) expression by gAcrp. Furthermore, decrease in Bcl‐2 mRNA half‐life due to gAcrp was also recovered by knockdown of TTP and AUF1 gene silencing (Fig. 3I, half‐life by treatment with gAcrp was 2.49 h, 4.87 h by gAcrp plus TTP gene silencing, and 3.91 h by gAcrp plus AUF1 gene silencing). Collectively, these results suggest that both TTP induction and AUF1 induction contribute to the suppression of Bcl‐2 expression by gAcrp via mRNA destabilization.

### TTP induction and AUF1 induction are implicated in gAcrp‐induced apoptosis and decrease in cell viability of HepG2 cells

**Figure 4:**
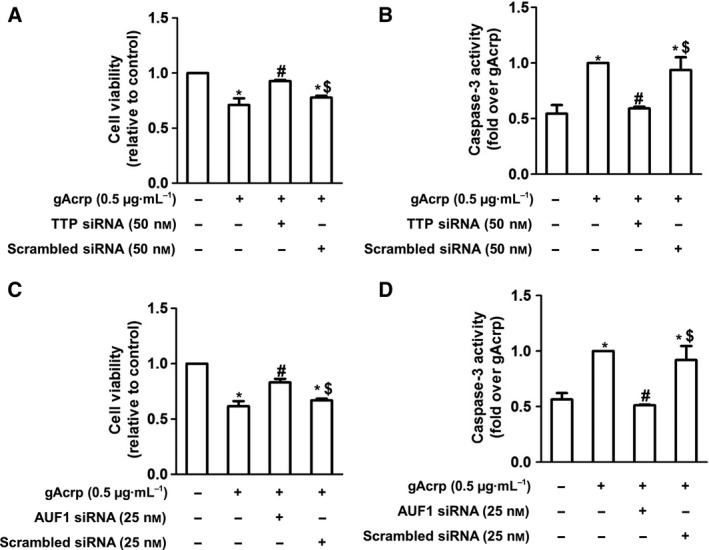
Role of TTP and AUF1 induction in the decrease in HepG2 cell viability and apoptosis by gAcrp. HepG2 cells were transfected either with TTP siRNA, AUF1 siRNA, or scrambled control siRNA. After 36‐h incubation, cells were then treated with gAcrp (0.5 μg·mL^−1^) for 24 h. Cell viability (A, C) and caspase‐3 activity (B, D) were then measured by MTS assay and caspase‐3 activity assay, respectively. Values represent fold change relative to the control cells and are presented as mean ± SEM (*n* = 3–4). *P < 0.05 as compared with the control cells; ^**#**^ *P* < 0.05 as compared to the cells treated with gAcrp, but not transfected; ^$^ *P* < 0.05 as compared to the cells treated with gAcrp and transfected with TTP siRNA or AUF1 siRNA. In all graphs, data were analyzed by one‐way ANOVA, followed by Tukey's *post hoc* test to compare multiple groups by graph prism software.

We next examined the functional roles of TTP and AUF1 induction in the suppression of tumor growth by gAcrp. As shown in Fig. 4, gene silencing of TTP or AUF1 by transfection with siRNA resulted in restoration of the gAcrp‐induced decrease in cell viability of HepG2 cells (Fig. 4A,C). Furthermore, caspase‐3 activation by gAcrp was prominently blocked by transfection with siRNA targeting TTP (Fig. 4B) or AUF1 (Fig. 4D). Overall, these results suggest that induction of TTP and AUF1 is implicated in gAcrp‐induced apoptotic cell death of HepG2 cells.

### Both adiponectin receptor type 1 signaling and type 2 signaling mediate Bcl‐2 mRNA destabilization and suppression of hepatic cancer cell growth by gAcrp

**Figure 5:**
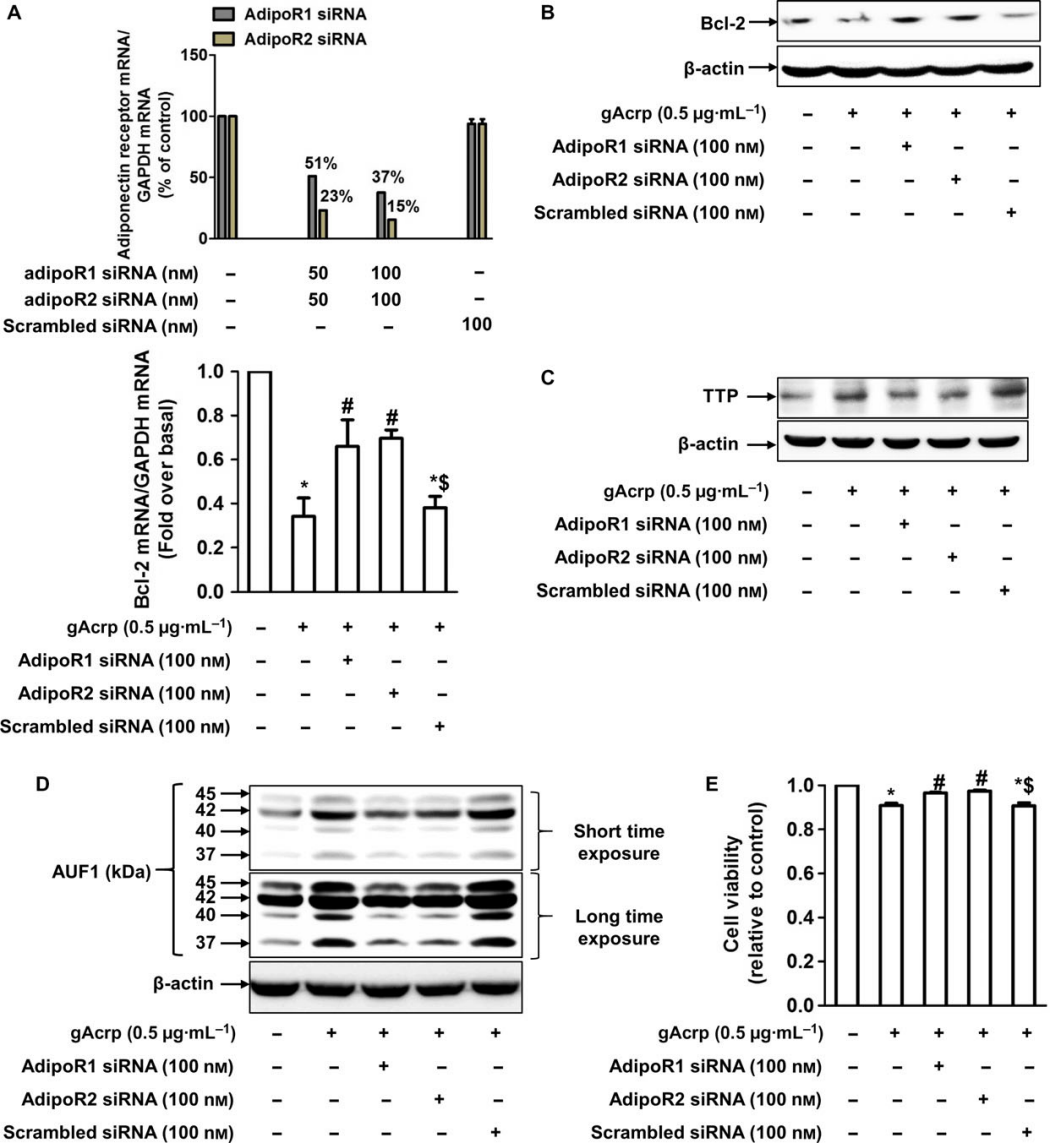
Role of adipoR1 and adipoR2 in TTP and AUF1 induction, suppression of Bcl‐2 expression, and cell viability by gAcrp in HepG2 cells. HepG2 cells were transfected with siRNA targeting adipoR1, adipoR2, or scrambled control siRNA. Gene‐silencing efficiency of adipoR1 and adipoR2 was monitored by qRT‐PCR (upper panel of A). After 36 h of transfection, cells were treated with gAcrp (0.5 μg·mL^−1^) for 24 h. (A) Bcl‐2 mRNA expression level was measured by qRT‐PCR. Data were analyzed by one‐way ANOVA, followed by Tukey's *post hoc* test, and values are expressed as mean ± SEM (*n* = 4). *P < 0.05 as compared with the control cells; ^#^ *P* < 0.05 as compared with the cells treated with gAcrp; ^$^ *P* < 0.05 compared to the cells treated with gAcrp and transfected with adipoR1 siRNA or adipoR2 siRNA. (B) Bcl‐2 protein expression level was measured by western blot analysis. (C) TTP protein expression level was determined by western blot analysis. (D) AUF1 protein expression level was measured by western blot analysis. In the western blot analyses, β‐actin was used as an internal loading control. Representative images from three independent experiments are shown. (E) Cell viability was determined by MTS assay. One‐way ANOVA, followed by Tukey's *post hoc* test, was used to analyze all data and values are indicated as fold changes relative to the control cells and expressed as mean ± SEM (*n* = 4). *P < 0.05 as compared with the control cells; ^#^ *P* < 0.05 as compared with the cells treated with gAcrp, but not transfected; ^$^ *P* < 0.05 compared to the cells treated with gAcrp and transfected with adipoR1 siRNA or adipoR2 siRNA.

Adiponectin‐induced physiological responses are initiated by binding to adiponectin receptor type 1 (adipoR1) and type 2 (adipoR2). In a series of experiments to identify the specific receptor type involved, gene silencing of both adipoR1 and adipoR2 substantially restored gAcrp‐induced decrease in Bcl‐2 mRNA expression (Fig. 5A). Suppression of Bcl‐2 protein expression was also restored to almost normal levels by knockdown of adipoR1 or adipoR2 (Fig. 5B). Moreover, induction of TTP and AUF1 by gAcrp was significantly suppressed by silencing adipoR1 or adipoR2 (Fig. 5C,D), suggesting that both adipoR1 signaling and adipoR2 signaling mediate Bcl‐2 mRNA destabilization by gAcrp via TTP and AUF1 induction. Finally, we observed that decrease in cell viability of HepG2 cells by gAcrp was markedly restored by silencing adipoR1 or adipoR2 (Fig. 5E), clearly indicating that both adipoR1 signaling and adipoR2 signaling play roles in modulation of hepatic cancer cell growth by gAcrp.

## Discussion

Accumulating evidence indicates that adiponectin plays a modulatory role in the development and progression of various tumors 3, 17. The antitumor activity of adiponectin is mediated by various mechanisms, and recent studies have revealed that adiponectin induces apoptosis in cancer cells by regulating the expression of anti‐ and pro‐apoptotic genes. In particular, adiponectin suppresses expression of Bcl‐2, an anti‐apoptotic gene, in cancer cells 18, 19. Given the previous reports that Bcl‐2 plays a critical role in modulating mitochondria‐mediated apoptosis via interaction with pro‐apoptotic genes, suppression of Bcl‐2 expression might be one of the key mechanisms contributing to antitumor activity of adiponectin. However, molecular mechanisms underlying suppression of Bcl‐2 expression are largely unknown. In the present study, we have demonstrated for the first time that gAcrp induces Bcl‐2 mRNA destabilization in hepatic cancer cells. In addition, TTP induction and AUF1 induction play a crucial role in Bcl‐2 mRNA destabilization and suppression of hepatic cancer cell growth by gAcrp. Herein, we also observed that gAcrp significantly decreased cancer cell growth and significantly increased caspase‐7 enzyme activity (Fig. S1), caused TTP and AUF1 induction, and suppressed BCl‐2 expression (Fig. S2) in MCF‐7 breast cancer cells with a pattern similar to that observed in hepatic cancer cells. Although we did not conduct all the experiments in MCF‐7 cells, these results further suggest that molecular mechanisms proposed in this study for inhibition of tumor growth by gAcrp might apply to breast cancer cells and to other different types of tumors.

As indicated earlier, it is well established that adiponectin possesses potent antitumor activities and causes cell death in various types of cancer cells. Interestingly, in contrast to this notion, previous studies have indicated that adiponectin also plays cytoprotective roles against diverse cytotoxic stimuli. For example, gAcrp protects hepatocytes from tunicamycin‐induced cell death by suppressing activation of inflammasomes 20 and supports colon cancer cell survival in the absence of glucose by augmenting autophagy machinery 21. In addition, under insulin deficiency, adiponectin improves systemic metabolism and promotes reconstitution of β‐cell mass in the mouse model 22, indicating that modulatory effects of adiponectin on cell death/survival would be depending on physiological conditions. In general, adiponectin is prone to protect cells from cellular damages induced by cytotoxic stimuli, whereas treatment with adiponectin alone generates cytotoxic effects on cancer cells. This kind of modulation by adiponectin can be observed in other physiological responses. For example, gAcrp suppresses the expression of endotoxin‐stimulated inflammatory mediators in immune cells 23, whereas adiponectin alone increases the expression of inflammatory mediators in macrophages 24, 25. Currently, understanding of the mechanisms underlying differential regulation of adiponectin in cell death and survival is limited. Further studies would provide further insights into how adiponectin modulates cell death and/or survival in different cellular environments.

A growing body of evidence indicates that Bcl‐2 interferes with programmed cell death independent of cell division 26, 27. Overexpression of Bcl‐2 in the presence of interleukin‐3 (IL‐3), acting as a growth factor, caused proliferation of lymphoid and myeloid cells. However, in the absence of IL‐3, cells survived in the G_0_ state in the presence of Bcl‐2, but did not proliferate 28, suggesting that Bcl‐2 acts within the ‘survival signal pathway’. Herein, we observed that gAcrp inhibited Bcl‐2 expression in HepG2 cells (Fig. 1E–H), which is accompanied with induction of caspase‐3 activity (Fig. 1C,D), suggesting that gAcrp induces apoptotic cell death triggered by suppression of Bcl‐2 in cancer cells. Bcl‐2 expression is regulated by various mechanisms. For example, there is a growing appreciation that Bcl‐2 expression is post‐translationally regulated via 26S proteasome‐mediated ubiquitination and degradation 29. However, in the present study, we found that inhibition of proteasome degradation process by pretreatment with MG‐132 did not restore suppression of Bcl‐2 expression by gAcrp (Fig. 2A). Moreover, gAcrp treatment did not significantly affect Bcl‐2 promoter activity (Fig. 2B), collectively indicating that suppression of Bcl‐2 expression by gAcrp is not regulated by proteasomal degradation or at transcriptional level. We finally speculated whether gAcrp suppresses Bcl‐2 expression by regulating the stability of Bcl‐2 mRNA. Once transcribed, mRNA is targeted and modulated by various mechanisms. For example, newly synthesized mRNA is degraded by decapping enzymes 30. In addition, microRNAs form a complementary bond with the target mRNA and induce post‐transcriptional silencing or block its translation 31. Moreover, the stability of mRNA is modulated by mRNA‐binding proteins 11, 12. Of the various mechanisms involved, we examined the role of mRNA‐binding proteins in the modulation of Bcl‐2 mRNA stability. Previous studies have demonstrated that Bcl‐2 mRNA contains AREs at 3′‐UTR and its stability is regulated by a number of mRNA‐binding proteins 32. In the present study, we demonstrated that gAcrp decreased Bcl‐2 mRNA half‐life and induced an increase in TTP and AUF1 expression, which act as mRNA‐destabilizing proteins. Furthermore, gene silencing of TTP or AUF1 restored decrease in Bcl‐2 mRNA half‐life (Fig. 3I) and prevented suppression of Bcl‐2 expression (Fig. 3C,D,G,H) by gAcrp, clearly indicating the crucial roles of TTP and AUF1 in the suppression of Bcl‐2 expression in hepatic cancer cells. Importantly, knockdown of TTP or AUF1 also restored gAcrp‐induced decrease in cell viability of hepatic cancer cells, implying that induction of TTP and AUF1 plays a key role in the modulation of tumor growth by gAcrp. A recent study reported that gAcrp decreases Bcl‐2 expression in macrophages through TTP induction, which contributes to autophagy induction by inhibiting interaction between Bcl‐2 with Beclin‐1, an autophagy‐activating protein. 15. Moreover, TTP induction contributes to the suppression of inflammatory mediators’ expression by adiponectin. In this study, we further demonstrated that AUF1, as well as TTP, is involved in Bcl‐2 mRNA destabilization in cancer cells. Furthermore, we demonstrated for the first time that Bcl‐2 mRNA destabilization by TTP and AUF1 plays a functional role in the suppression of tumor growth by adiponectin. To the best of our knowledge, this is the first report to demonstrate that adiponectin induces Bcl‐2 mRNA destabilization in cancer cells and induction of TTP and AUF1 plays a pivotal role in the suppression of tumor growth by adiponectin.

A number of biological functions of adiponectin are mediated through binding with its specific receptors, mainly adiponectin receptor type 1 (adipoR1) and type 2 (adipoR2). While adipoR1 and adipoR2 generate similar metabolic effects, their effects are mediated by different signaling molecules. For example, adenosine monophosphate‐activated protein kinase (AMPK) plays a central role in mediating adpoR1 signaling, whereas adipoR2 signaling is mainly mediated by peroxisome proliferator‐activated receptor alpha 33. Moreover, different forms of adiponectin, that is, globular and full‐length adiponectin, have differential binding affinity for specific types of receptors. Globular adiponectin has a higher binding affinity for adipoR1 than for adipoR2 34, 35, and adipoR1 signaling plays a predominant role in anti‐inflammatory responses of gAcrp in macrophages 15. In contrast, adiponectin has been shown to induce apoptotic cell death of endometrial carcinoma through both adipoR1 and adipoR2 signaling, in which both adiponectin receptors involve AMPK pathway for induction of apoptosis 4, suggesting that involvement of a specific type of adiponectin receptor would be depending on experimental conditions. In the present study, we observed that transfection of siRNA targeting both adipoR1 and adipoR2 inhibited gAcrp‐induced increase in TTP and AUF1 induction, and restored suppression of Bcl‐2 expression caused by gAcrp (Fig. 5), suggesting that both adipoR1 and adipoR2 signaling might be involved in suppressing the growth of hepatic cancer cells caused by gAcrp. The results presented in this study are different from those observed in macrophages and confirm that adiponectin receptor signaling is modulated depending on experimental conditions.

Herein, we have examined the effects of transfection of siRNA targeting TTP and AUF1 on basal levels of Bcl‐2 mRNA stability, caspase‐3 activity, and cell viability in HepG2 hepatic cancer cells. We observed that TTP or AUF1 siRNA increased cell viability (Fig. S4A). It was a modest effect (around 10%), but consistent and statistically significant. In addition, TTP and AUF1 siRNA also decreased activity of caspase‐3 at basal level (Fig. S4B). Similar to the results from cell viability assay, inhibitory effect on caspase‐3 activity was modest, but also consistent and statistically significant without significant effects by transfection with scrambled siRNA. Furthermore, we have found that transfection with siRNA targeting TTP or AUF1 slightly increased Bcl‐2 mRNA half‐life (Bcl‐2 mRNA half‐life at basal level was 4.34 h versus TTP siRNA and AUF1 siRNA was 5.95 and 5.39 h, respectively; Fig. S4C). Taken together, all these results consistently indicate that TTP and AUF1 are implicated in apoptosis induction (caspase‐3 activation) and subsequent decrease in cell viability in HepG2 cells at basal level by inducing Bcl‐2 mRNA destabilization. The modulatory effects of TTP and AUF1 siRNA on basal levels of Bcl‐2 mRNA stability, caspase‐3 activity, and cell viability were very modest compared with those from the cells stimulated with gAcrp (Figs 3 and 4). As indicated earlier, adiponectin treatment induced significant increase in AUF1 and TTP expression, and modulation of Bcl‐2 mRNA stability is mediated by induction of AUF1 and TTP. Therefore, modulatory effects by AUF1 and TTP siRNA would be more prominent in the cells treated with gAcrp. At this stage, we could not thoroughly address the roles of AUF1 and TTP in modulating apoptosis and growth of cancer cells, it appears that AUF1 and TTP are implicated in the modulation of tumor growth by regulating Bcl‐2 expression even at basal level. Further studies would provide further insights into the role of AUF1 and TTP in the regulation of tumor growth.

In conclusion, the data presented in this study demonstrate for the first time that the suppressive effect of gAcrp on tumor growth is mediated via destabilization of Bcl‐2 mRNA. In addition, induction of AUF1 and TTP contributes to Bcl‐2 mRNA destabilization. Based on the data presented here and in previous reports, we suggest that AUF1 and TTP are novel putative targets implicated in the suppression of tumor growth by adiponectin. Further studies validating the role of these mRNA‐binding proteins *in vivo* are required to understand the mechanisms underlying the antitumor activity of adiponectin.

## Author contributions

P‐HP, SHK, and NTP designed the study. NTP, AS, and AK performed experiments. P‐HP, SHK, NTP, and AK analyzed the data. P‐HP and NTP wrote the manuscript.

## Conflict of interest

The authors declare no conflict of interest.

## Supporting information


**Fig. S1.** Effect of gAcrp on cell viability in MCF‐7 cells. (A) MCF‐7 cells were treated with gAcrp (0.5 μg·mL^−1^) for the indicated time durations. Cell viability was measured by MTS assay. Data were analyzed by ANOVA, followed by Tukey's *post hoc* multiple comparison test and values are presented as the fold increase in comparison to the control cells and expressed as mean ±** **SEM (*n* = 3). *P < 0.05 as compared to control cells. (B) MCF‐7 cells were treated with gAcrp (0.5 μg·mL^−1^) for the indicated time durations. Caspase‐7 activity was determined using caspase‐Glo3/7 activity assay kit (Promega) according to the manufacturer's instruction. One way ANOVA combined with Tukey's *post hoc* multiple comparison test was used to analyze data and values are presented as the fold increase in comparison to the control cells and expressed as mean ± SEM (*n* = 3). *P < 0.05 compared with control cells.

**Fig. S2.** Effect of gAcrp on TTP, AUF1 and Bcl‐2 protein expression in MCF‐7 cells. MCF‐7 cells were treated with gAcrp (0.5 μg·mL^−1^) for the indicated time durations. Protein expression levels of TTP, AUF1, and Bcl‐2 were measured by Western blot analyses. Representative images from 2 sets of independent experiments that showed the same results are shown along with β‐actin as an internal loading control.

**Fig. S3.** Effect of gAcrp on cell viability in HepG2 cells. (A) HepG2 cells were treated with gAcrp (0.5 μg·mL^−1^) for the indicated time durations. Cell viability was measured by MTS assay. One way ANOVA followed by Tukey's *post hoc* test was used to analyze the data and values are presented as the fold increase in comparison to the control cells and expressed as mean ±** **SEM (*n* = 3). *P < 0.05 as compared to control cells.

**Fig. S4.** Effects of TTP and AUF1 siRNA on basal levels of cell viability, caspase‐3 activity, and Bcl‐2 mRNA half‐life in HepG2 cells. (A) HepG2 cells were transfected with siRNA targeting TTP or AUF1 for 36 h. Cell viability was measured by MTS assay. Values are presented as the fold increase in comparison to the control cells and expressed as mean ± SEM (*n* = 3). *P < 0.05 as compared to control cells. (B) HepG2 cells were transfected with siRNA targeting TTP or AUF1 for 36 h. Caspase‐3 activity was determined using Caspase‐3 activity assay kit. Values are presented as the fold increase in comparison to the control cells and expressed as mean ± SEM (*n* = 3). *P < 0.05 as compared to control cells. In both A and B, data were analyzed by One way ANOVA followed by Tukey's *post‐hoc* test for multiple comarison. (C) HepG2 cells were transfected with siRNA targeting TTP or AUF1. After 36 h, the cells were treated with actinomycin D (2 μg·mL^−1^) for 3, 8, or 12 h. Accumulating Bcl‐2 mRNA level was measured by qRT‐PCR and used for calculation of half‐life. Percentage remaining of Bcl‐2 mRNA was calculated as % of control (mean ± SEM, *n* = 3). Half‐life calculation and statistical significance were determined by sigma plot software version using equation First Parameter Logistic obtained by linear regression of plot from mRNA percentage remaining (%) and standard deviation.
