# Supplementary material for: Critical role of tristetraprolin and AU‐rich element RNA‐binding protein 1 in the suppression of cancer cell growth by globular adiponectin
Source: FEBS Open Bio. 2018 Nov 12;8(12):1964–76. doi: 10.1002/2211-5463.12541 (PMC6275284; doi:10.1002/2211-5463.12541)
Supplement: Supplementary file 5 [file FEB4-8-1964-s005.docx]

**Supplementary Figure 1. Effect of gAcrp on cell viability in MCF-7 cells**

(A) MCF-7 cells were treated with gAcrp (0.5 μg/ml) for the indicated time durations. Cell viability was measured by MTS assay. Data were analyzed by ANOVA, followed by Tukey’s post hoc multiple comparison test and values are presented as the fold increase in comparison to the control cells and expressed as mean **±** SEM (n = 3). *P < 0.05 as compared to control cells. (B) MCF-7 cells were treated with gAcrp (0.5 μg/ml) for the indicated time durations. Caspase-7 activity was determined using caspase-Glo3/7 activity assay kit (Promega) according to the manufacturer’s instruction. One way ANOVA combined with Tukey’s post hoc multiple comparison test was used to analyze data and values are presented as the fold increase in comparison to the control cells and expressed as mean **±** SEM (n = 3). *P < 0.05 compared with control cells.

**Supplementary Figure 2. Effect of gAcrp on TTP, AUF1 and Bcl-2 protein expression in MCF-7 cells**

MCF-7 cells were treated with gAcrp (0.5 μg/ml) for the indicated time durations. Protein expression levels of TTP, AUF1, and Bcl-2 were measured by Western blot analyses. Representative images from 2 sets of independent experiments that showed the same results are shown along with β-actin as an internal loading control.

**Supplementary Figure 3. Effect of gAcrp on cell viability in HepG2 cells**

(A) HepG2 cells were treated with gAcrp (0.5 μg/ml) for the indicated time durations. Cell viability was measured by MTS assay. One way ANOVA followed by Tukey’s post hoc test was used to analyze the data and values are presented as the fold increase in comparison to the control cells and expressed as mean **±** SEM (n = 3). *P < 0.05 as compared to control cells.

**Supplementary Figure 4. Effects of TTP and AUF1 siRNA on basal levels of cell viability, caspase-3 activity, and Bcl-2 mRNA half-life in HepG2 cells**

(A) HepG2 cells were transfected with siRNA targeting TTP or AUF1 for 36 h. Cell viability was measured by MTS assay. Values are presented as the fold increase in comparison to the control cells and expressed as mean **±** SEM (n = 3). *P < 0.05 as compared to control cells. (B) HepG2 cells were transfected with siRNA targeting TTP or AUF1 for 36 h. Caspase-3 activity was determined using Caspase-3 activity assay kit. Values are presented as the fold increase in comparison to the control cells and expressed as mean **±** SEM (n = 3). *P < 0.05 as compared to control cells. In both A and B, data were analyzed by One way ANOVA followed by Tukey’s post-hoc test for multiple comarison. (C) HepG2 cells were transfected with siRNA targeting TTP or AUF1. After 36 h, the cells were treated with actinomycin D (2 μg/ml) for 3 h, 8 h, or 12 h. Accumulating Bcl-2 mRNA level was measured by qRT-PCR and used for calculation of half-life. Percentage remaining of Bcl-2 mRNA was calculated as % of control (mean **±** SEM, n = 3). Half-life calculation and statistical significance were determined by sigma plot software version using equation First Parameter Logistic obtained by linear regression of plot from mRNA percentage remaining (%) and standard deviation.
